# Supplementary material for: Exploiting the tunability of stimulated emission depletion microscopy for super-resolution imaging of nuclear structures
Source: Nat Commun. 2018 Aug 24;9:3415. doi: 10.1038/s41467-018-05963-2 (PMC6109149; doi:10.1038/s41467-018-05963-2)
Supplement: Supplementary file 1 — Supplementary Information [file 41467_2018_5963_MOESM1_ESM.pdf]

Supporting Info for:

Exploiting the tunability of stimulated emission depletion microscopy for super-resolution imaging of nuclear structures

Sarmento et al

Supplementary Figures 1-16

Supplementary Note 1

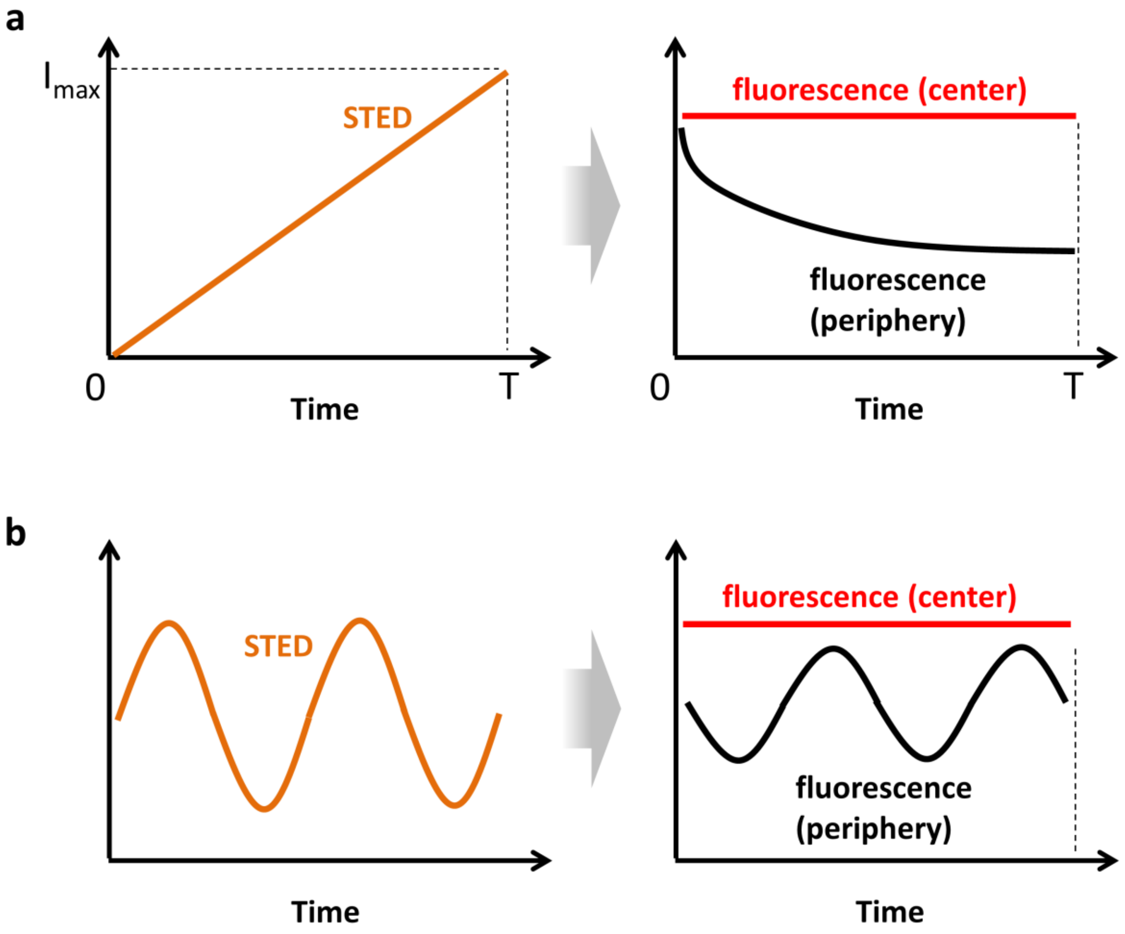

Supplementary Fig.1. Modulation of the STED intensity in time. (a) Schematic of the modulation pattern used in this work. The STED intensity was linearly increased from zero to a maximum I_{max} within a time interval T. As a consequence, while fluorescence emission from the center of the PSF remains constant (since it coincides with the center of the STED donut where I_{STED}=0), in the periphery the emission decreases in a spatially-dependent manner. (b) Schematic of a sinusoidal modulation of the STED intensity. In this case, fluorescence in the center also remains constant, unaffected by the STED, whereas fluorescence in the periphery is modulated at the same frequency.

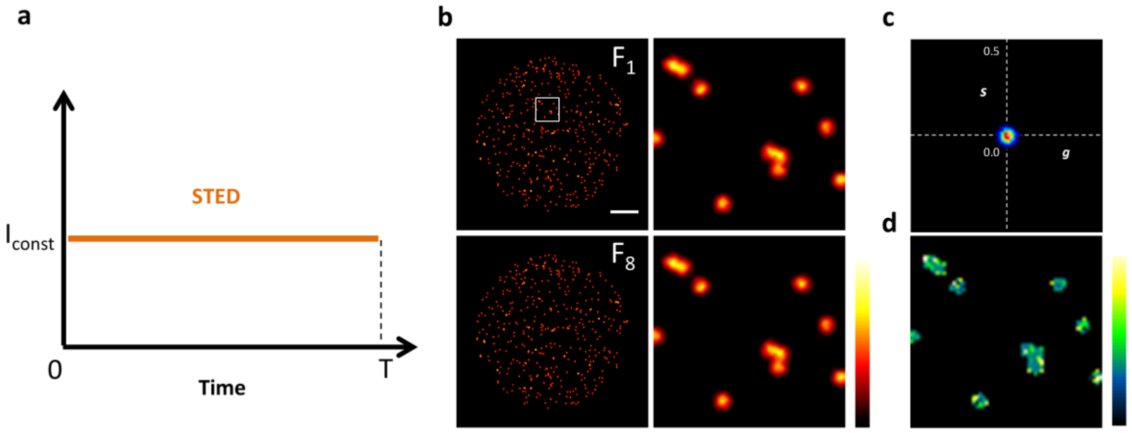

Supplementary Fig.2. Simulation of an M-STED stack at constant STED intensity. (a) Schematic of the implementation in time. (b) Simulation of nuclear foci. An eight-image stack was simulated from F_1 to F_8 at constant STED intensity I_{const} throughout the stack. Color scale: normalized intensity. Scale bar: 3 µm. (c) Phasor representation of fluorescence emission simulated in b. (d) Respective modulation image *M(x,y)*. Since there is no depletion dynamics, the phasor appears at the position (0,0) and the *M(x,y)* does not show any patterned modulation. Color scale: modulation *M* (0-0.13).

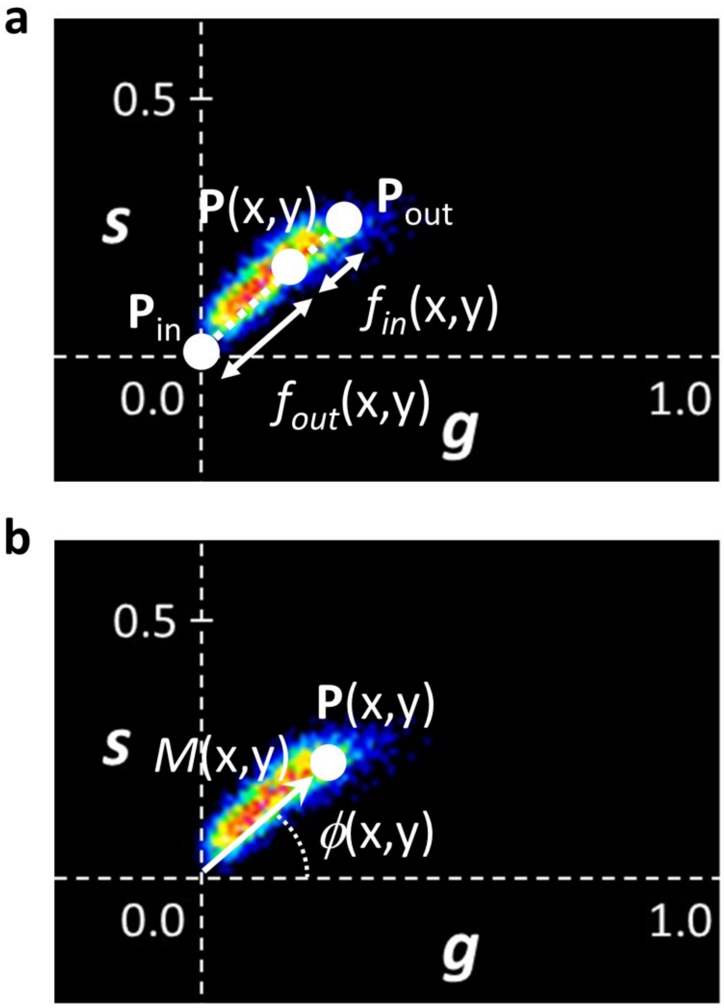

Supplementary Fig.3. Phasor analysis of the data. (a) Calculation of the fractional contribution $f_{in}(x,y)$ and $f_{out}(x,y)$, corresponding to the center and to the periphery of the PSF. The fractions $f_{in}(x,y)$ and $f_{out}(x,y)$ are proportional to the distance between the phasor measured in a given pixel, $\mathbf{P}(x,y)$, and the phasor $\mathbf{P}_{out}$ and $\mathbf{P}_{in}$, respectively. (b) Representation of a phasor $\mathbf{P}(x,y)$ in the polar coordinates $M(x,y)$ and $\phi(x,y)$.

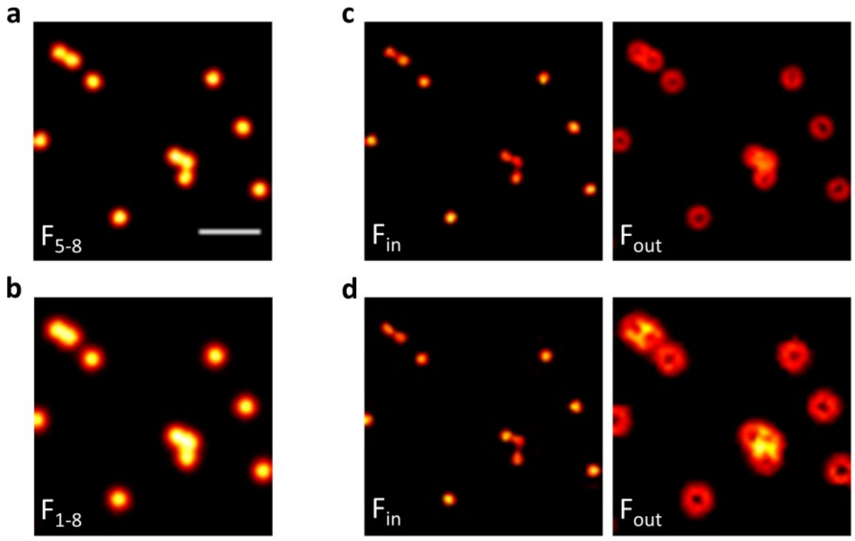

Supplementary Fig.4. SPLIT operation applied to a sum of the images of the stack simulated in Figure 1e of the main text. (a,b) Partial (F_5 to F_8) and total (F_1 to F_8) sum of the images within the eight-image stack were used for SPLIT. Scale bar: 0.6 µm. (c,d) SPLIT image components corresponding to the center (F_{in}) and to the periphery (F_{out}) of the PSF resulting from the application of SPLIT to a and b, respectively.

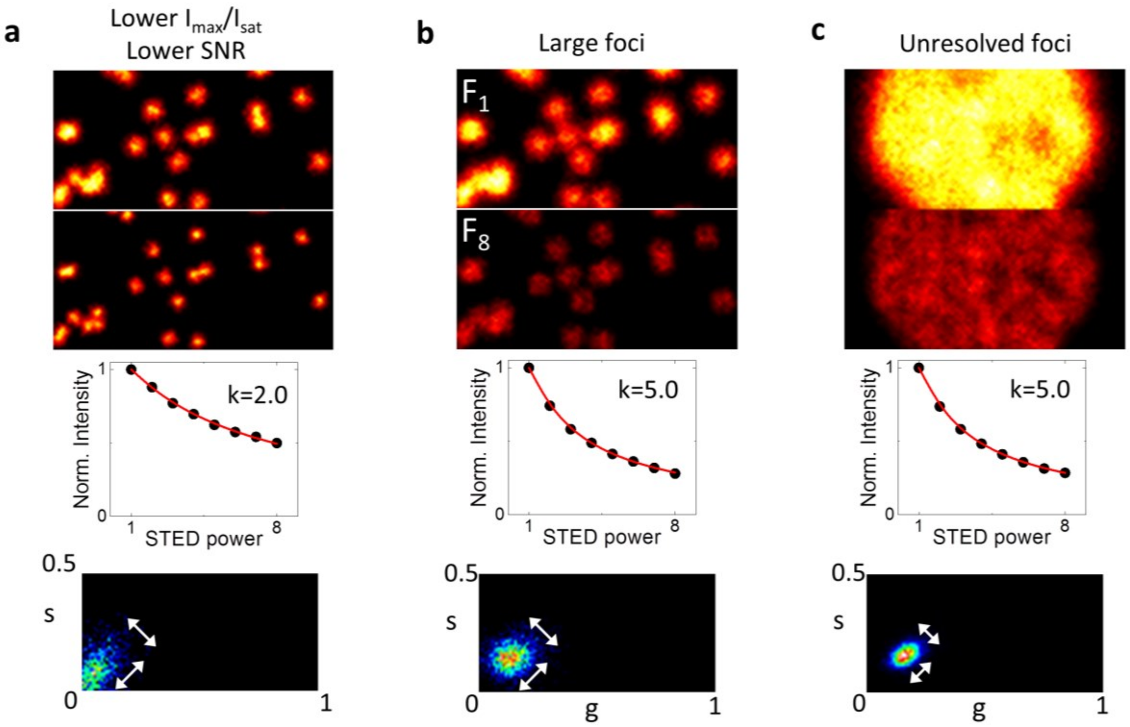

Supplementary Fig.5. Additional simulations of M-STED acquisitions. (a) Simulation of sparse foci acquired at lower maximum saturation value (I_{max}/I_{sat}=2) and lower SNR (S_{max}=10), compared with Figure 2a of the main text. (b,c) Simulations of large and unresolved foci, respectively, as described in the Methods section. Shown are, for all the simulations, from top to bottom: first and last images of the stack (F_1 and F_8), the average variation of fluorescence intensity as a function of the STED power and the estimated *k* parameter, and the corresponding phasor plot.

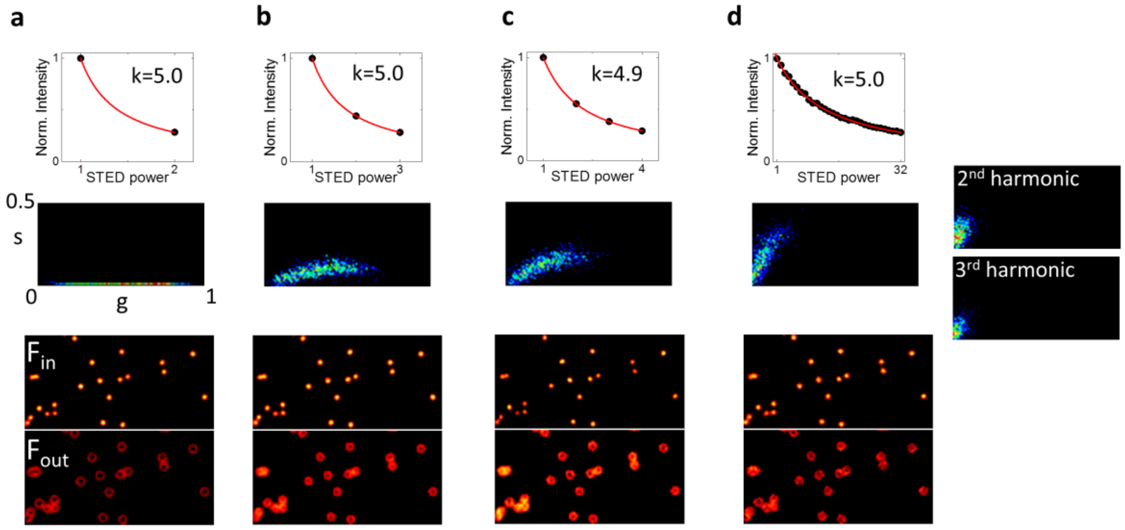

Supplementary Fig.6. Simulations of M-STED acquisitions with different number of STED powers within the stack (number of images, *n*). (a-d) Simulations of sparse foci using *n*=2 (a), *n*=3 (b), *n*=4 (c), *n*=32 (d). Shown are, from top to bottom, the depletion curve, the phasor plot and the SPLIT image components F_{in} and F_{out}. In (d) the phasor plots corresponding to the 2nd and 3rd harmonic are also shown.

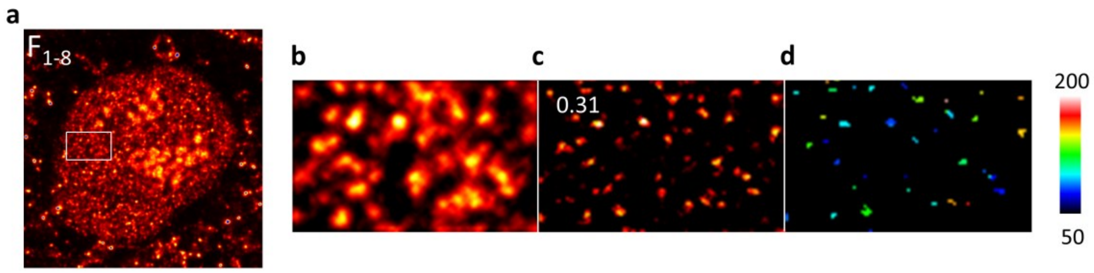

Supplementary Fig.7. Additional example of data used for estimating the size of transcription foci. (a) Full-size sum of stack images acquired at P_{max}=43.3 mW. (b) ROI selected for the analysis. (c) SPLIT image and respective PSF size relative to confocal (w/w_c). (d) Apparent size of the foci depicted in (c). Colormap in (d) represents size in nm.

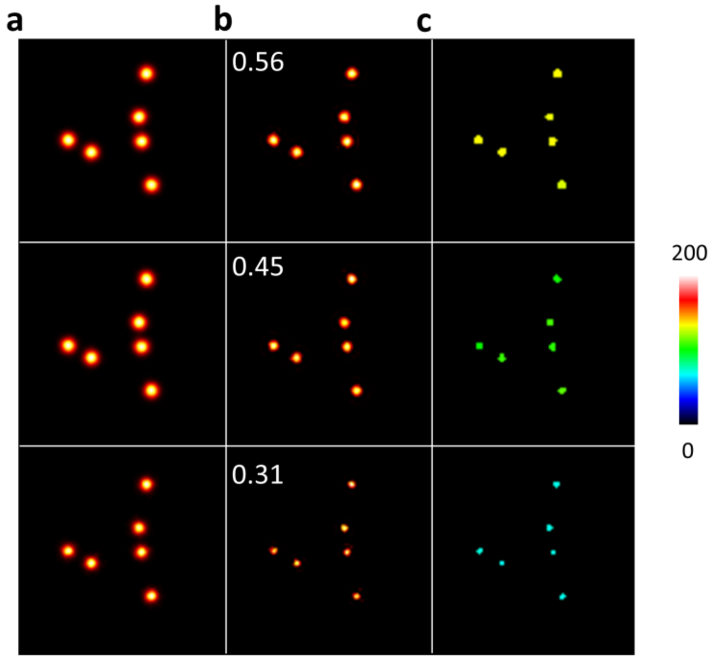

Supplementary Fig.8. Simulation of point-like structures and size quantification. (a) Examples of simulated M-STED images used for analysis; shown is the sum of the stack. (b) SPLIT images. Numbers indicate the estimated value of w/w_c. (c) Apparent size of simulated point-like objects. The square values of the average sizes retrieved from simulations at different w/w_c values are reported in Figure 3j of the main text. Colormap in (c) represents size in nm.

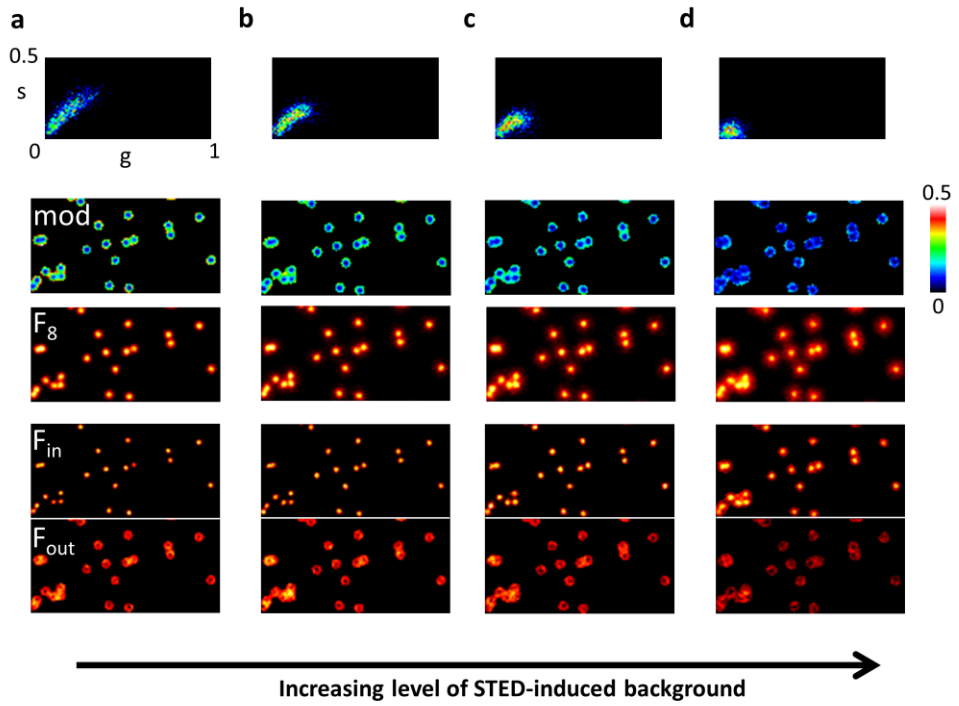

Supplementary Fig.9. Simulation of M-STED (original configuration) data at varying levels of STED-induced background. The value of background was set to B_{max}=0 (a), 5% (b), 10% (c), 20% (d), respectively. Shown are (from top to bottom) the phasor plot, the modulation image (mod), the last frame of stack (F_8) and the SPLIT components F_{in} and F_{out}.

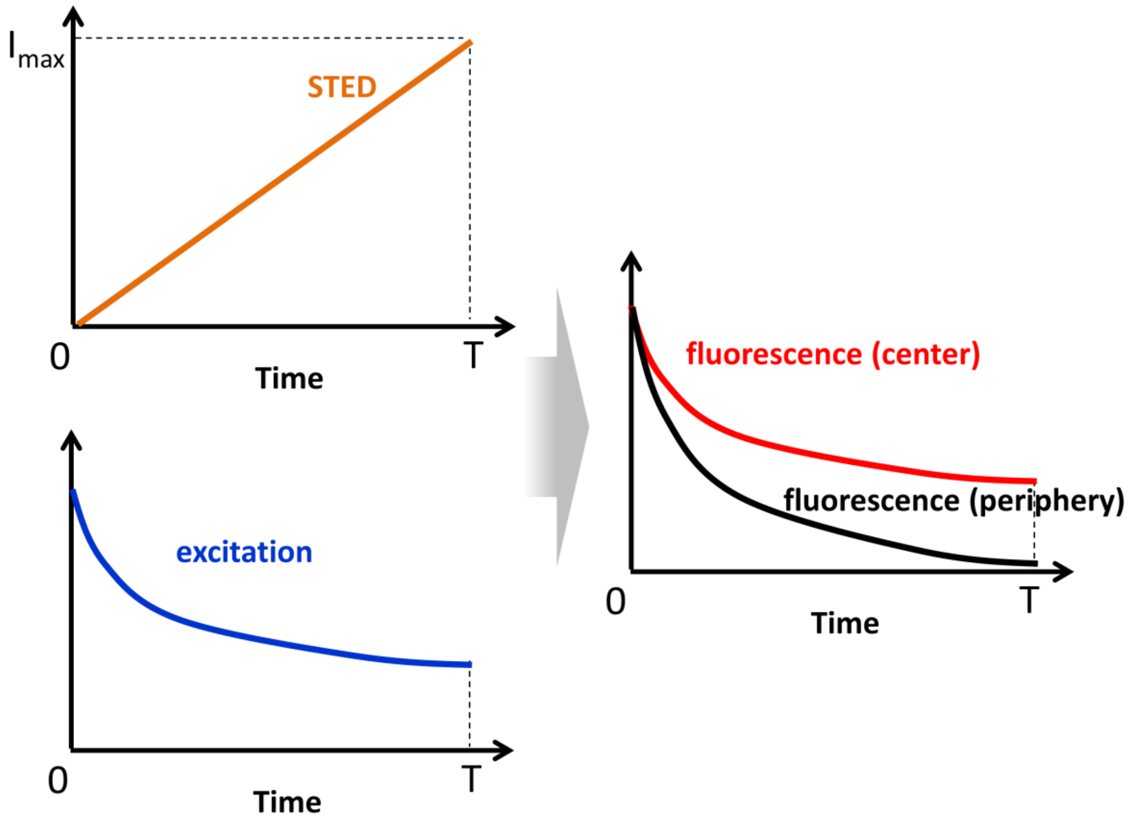

Supplementary Fig.10. Schematic of the modulation of both STED and excitation intensities in time. In the new configuration, while STED power is linearly increased up to $I_{\max}$, the excitation intensity is modulated as an exponential decay. Hence, in the center of the PSF, the fluorescence decays along the stack in a way that is dependent on the variation of the excitation power, while in the periphery the emission decay depends on both the excitation decay and the STED modulation.

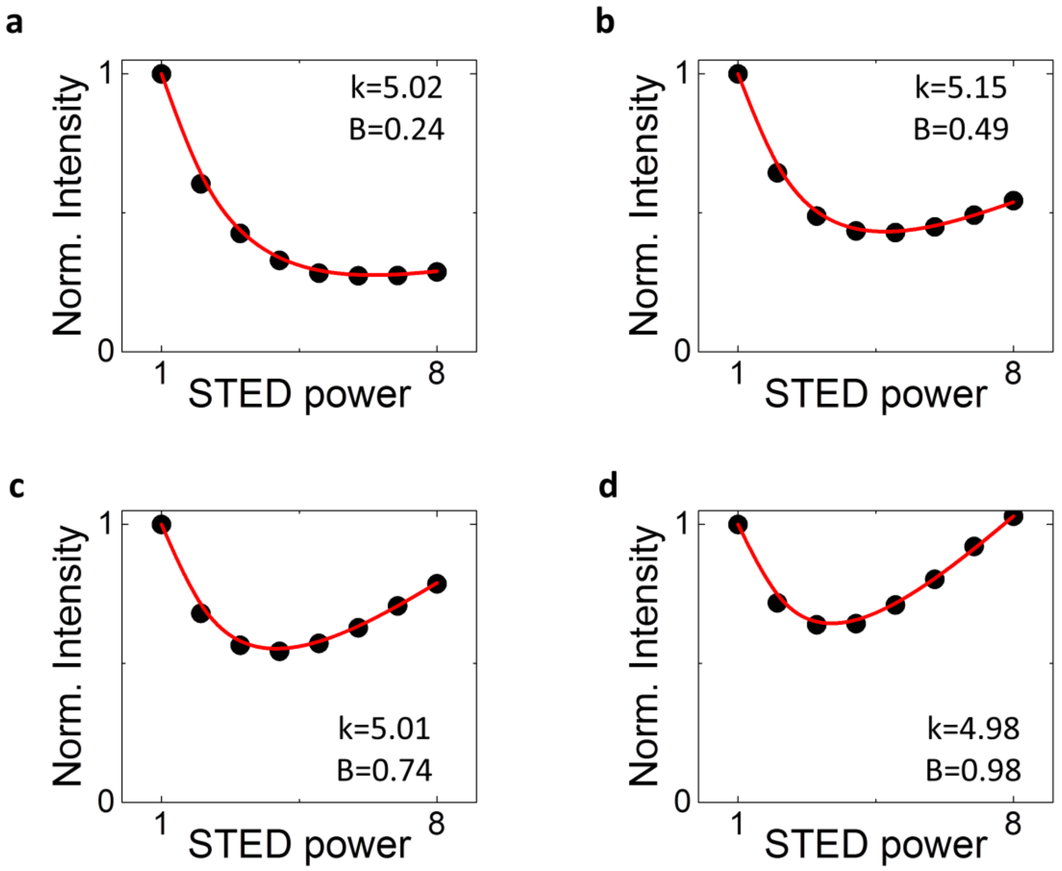

Supplementary Fig.11. Analysis of the average depletion curve in presence of STED-induced background. Eight-image stacks were simulated applying the modified version of M-STED with modulated excitation. The background parameter B_{max} was set to (a) 10%, (b) 20%, (c) 30% and (d) 40% of fluorescence emission. Eq. 6 was fitted to the data (average fluorescence intensity variation as a function of the STED power), to retrieve the depicted k and B parameters.

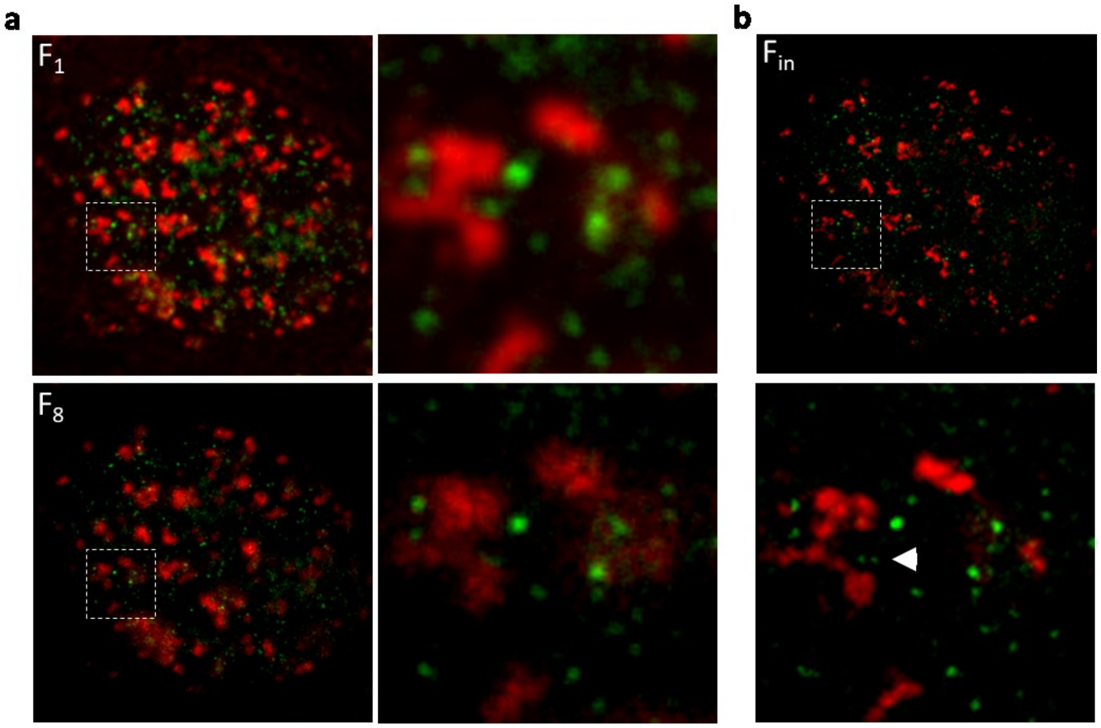

Supplementary Fig.12. Multi-color M-STED imaging of replication (red) and transcription (green) foci. (a) Example of 2-color M-STED stack showing the confocal F_1 image and the one at maximum STED power F_8 (P_{max}=24.2 mW). (b) F_{in} image resulting from the SPLIT analysis applied to both channels. The arrowhead points at two foci that are better resolved in the SPLIT image.

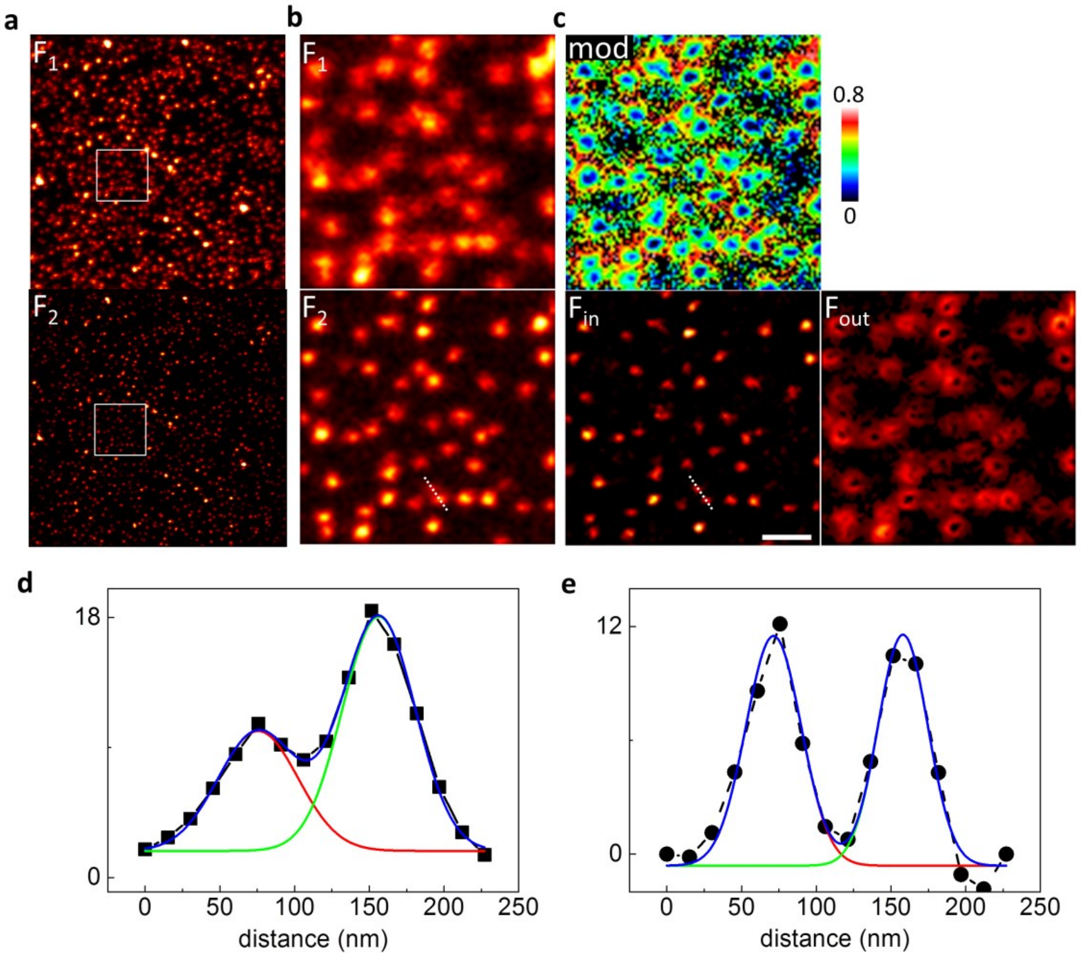

Supplementary Fig.13. M-STED imaging using two STED powers. (a,b) M-STED acquisition of a sample of 20 nm fluorescent beads. F_1 was acquired at P_1=33 mW and F_2 at P_2=125 mW. Time gating was set to 2 ns. (c) Modulation image and SPLIT image components. Scale bar: 300 nm. (d,e) Intensity profiles along the dashed line for the STED image (d) and the SPLIT image (e). Shown are the data and a multi-peak Gaussian fit. The values of FWHM extracted from the fits are $FWHM_1$=63 nm and $FWHM_2$=57 nm for the STED image and $FWHM_1$=43 nm and $FWHM_2$=39 nm for the SPLIT image.

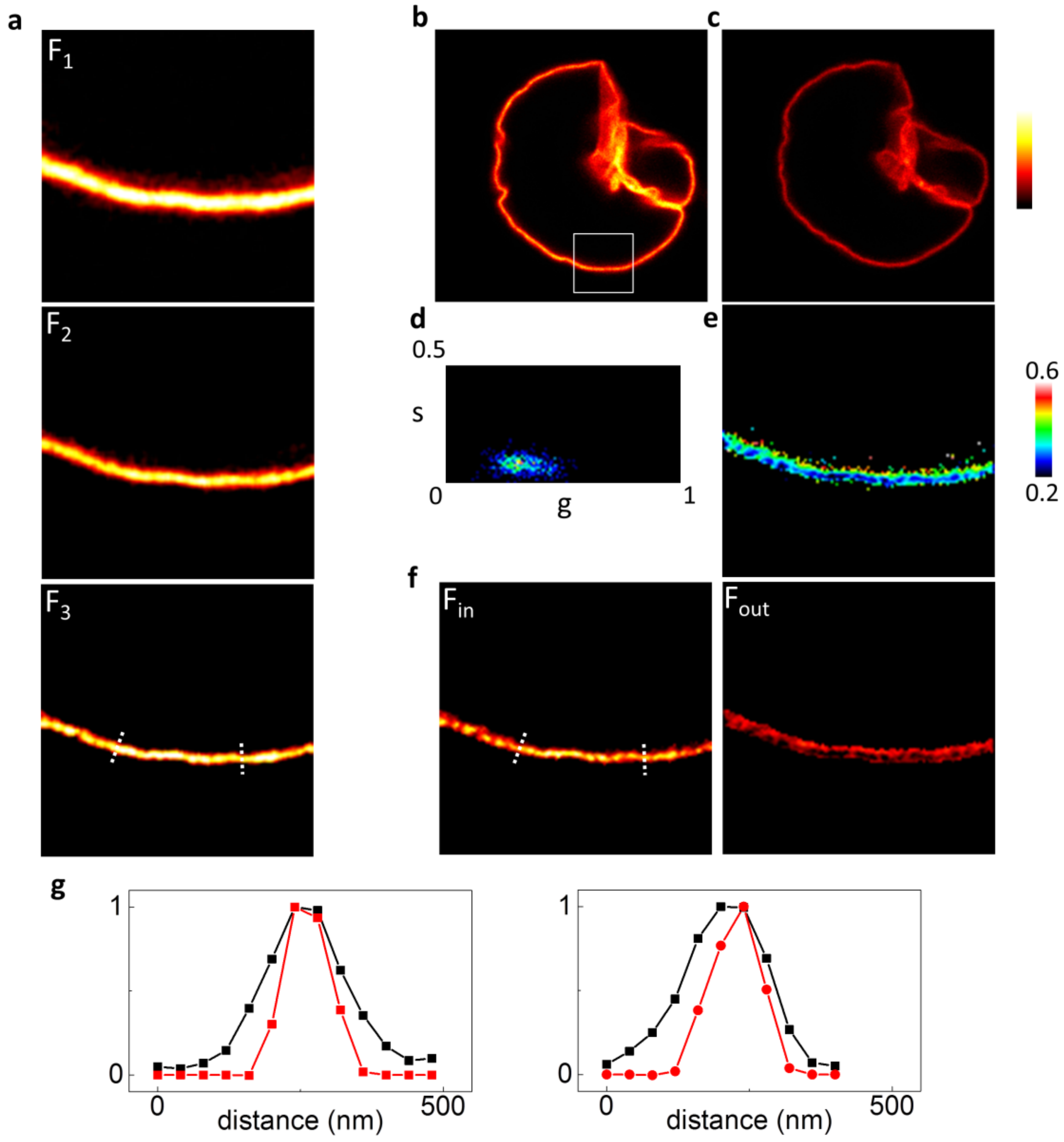

Supplementary Fig.14. M-STED imaging in live HEK-293 cells expressing GFP-Δ50 lamin A. (a) M-STED acquisition using three STED powers. F_1 was acquired at P_1=6 mW, F_2 at P_2=25 mW and F_3 at P_3=43 mW. Time gating was set to 2.5 ns. (b) Full size confocal image before M-STED acquisition. (c) Full size confocal image after M-STED acquisition. The intensity variation between (b) and (c) is due to photobleaching of GFP. (d,e,f) Phasor plot, modulation image and SPLIT image components. Image size in (b,c) is 20 μm. (g) Comparison between STED and SPLIT normalized intensity profiles along the dashed lines. The values of FWHM extracted from a Gaussian fit of the data (not shown) are $FWHM_{STED}$=153 nm and $FWHM_{SPLIT}$=92 nm for the first profile and $FWHM_{STED}$=160 nm and $FWHM_{SPLIT}$=105 nm for the second.

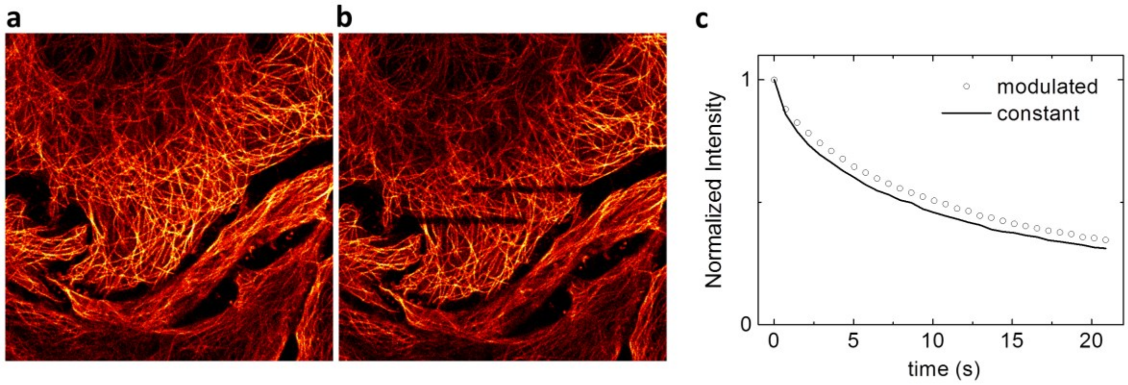

Supplementary Fig.15. Comparison of photobleaching in modulated and constant STED acquisition modes. Photobleaching was compared on the same cell by limiting the scanning to small rectangular areas and acquiring consecutive M-STED stacks with either modulated (P_{max}=24.2 mW) or constant STED (P_{const}=12.1 mW). (a,b) Image of microtubules in a fixed HeLa cell before (a) and after (b) photobleaching. (c) Normalized fluorescence intensity as a function of time. Intensity was calculated as the average intensity of each M-STED stack.

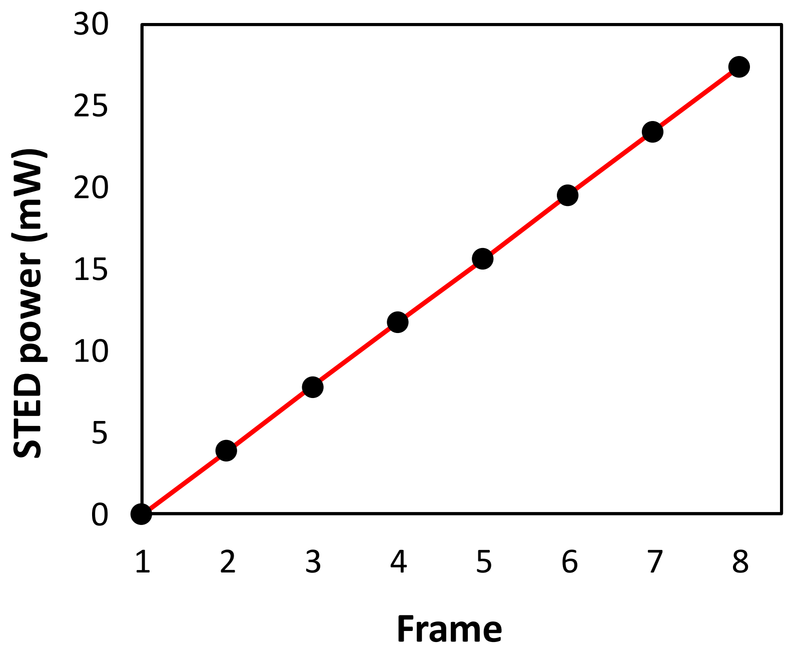

Supplementary Fig.16. Example of calibration of an M-STED ramp for n=8 and P_{max}=28 mW.

Supplementary Note 1 – Derivation of the expression for the average depletion curve

In [1] it was demonstrated that for the continuous distribution of decay rates described by:

$\gamma(r)=\gamma_0+\gamma_1 k_S r^2/w_c^2$

the average decay resulting from the convolution of single exponential decays exp(–$\gamma(r)t$) with a Gaussian PSF of waist w_c is given by:

$$\frac{\langle F(x,y,t)\rangle}{\langle F(x,y,0)\rangle}=e^{-\gamma_0 t}\frac{1}{1+\frac{k_S\gamma_1 t}{2}}$$

In M-STED we have:

$\gamma(r)=\gamma_0+(I_{max}/I_{sat})(r^2/w_c^2)/T$

Where γ_0=0 for constant excitation intensity along the stack and $\gamma_0=1/\tau_{exc}$ for excitation which is modulated as an exponential decay along the stack. In our implementation of M-STED, $T=n$-1 where n is the number of images forming the stack.

Equation 4 in the main text can be obtained by setting $\gamma_1 k_S=(I_{max}/I_{sat})/T$ and γ_0=0:

$$\frac{\langle F(x,y,t)\rangle}{\langle F(x,y,0)\rangle}=\frac{1}{1+\frac{k}{2}\frac{t}{T}}$$

And then substituting $t=(j$-1) and $T=(n$-1).

Similarly, equation 6 in the main text can be obtained by setting $\gamma_1 k_S=(I_{max}/I_{sat})/T$ and $\gamma_0=1/\tau_{exc}$:

$$\frac{\langle F(x,y,t)\rangle}{\langle F(x,y,0)\rangle}=e^{-\gamma_0 t}\frac{1}{1+\frac{k}{2}\frac{t}{T}}+B\frac{t}{T}$$

And then substituting $t=(j$-1) and $T=(n$-1).

Supplementary References

1. Lanzanò, L. *et al.* Encoding and decoding spatio-temporal information for super-resolution microscopy. *Nat. Commun.* **6,** 6701 (2015).
